# Primary Malignant Pulmonary Glomus Tumor: A Case Report and Literature Review

**DOI:** 10.1111/crj.70183

**Published:** 2026-03-22

**Authors:** Shishi Luo, Xiaoyan Lei, Tianxu Fu, Fujin Liu, Zhenping Wang

**Affiliations:** ^1^ Department of Radiology Hainan Affiliated Hospital of Hainan Medical University (Hainan General Hospital) Haikou Hainan China; ^2^ Department of Pathology Hainan Affiliated Hospital of Hainan Medical University (Hainan General Hospital) Haikou Hainan China; ^3^ Department of Radiology Hainan Hospital, Guangdong Provincial Hospital of Chinese Medicine Haikou Hainan China

**Keywords:** computed tomography, glomus tumor, malignant, pulmonary

## Abstract

Malignant glomus tumors originating from the lungs are relatively rare. We report a case of primary malignant pulmonary glomus tumor in a 41‐year‐old female. Chest CT revealed an 8 × 6 cm mass in the upper lobe of the right lung with multiple spotted calcifications, with uneven enhancement on enhanced scans; multiple metastatic nodules can be seen in both lungs; enlarged lymph nodes can be seen in the mediastinum and right hilar lobe. The pathological diagnosis of right lung puncture was malignant pulmonary glomus tumor. Chemotherapy and targeted therapy with arotinib hydrochloride were used, and survival was still observed after 36 months of follow‐up. The clinical manifestations of primary malignant pulmonary glomus tumor lack specificity. The imaging manifestations are well‐defined nodules or masses, with peripheral enhancement on enhanced scans. The imaging manifestations of benign and malignant pulmonary glomus tumor overlap with other lung tumors, making differential diagnosis difficult. The diagnosis is mainly based on pathology and immunohistochemical.

## Graphical abstract

We report a case of primary malignant pulmonary glomus tumor. A mass in the upper lobe of the right lung, with multiple speckled calcifications. The enhanced scan showed uneven enhancement in the arterial phase and progressive enhancement in the venous phase.

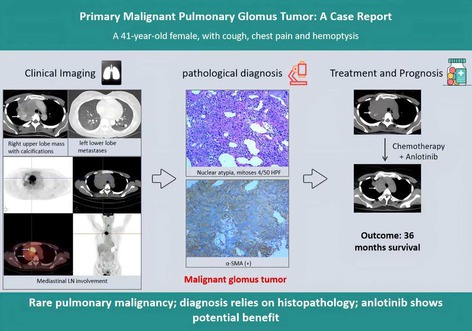

## 1 Introduction

Glomus tumor is a rare soft tissue mesenchymal tumor originating from perivascular glomus cells of arteriovenous anastomosis, accounting for about 1.6% of soft tissue tumors [1]. It was first reported by Masson [2]. Glomus tumors tend to occur in the dermis and subcutaneous tissues of extremities with abundant glomus, most of which are benign and the proportion of malignant glomus tumors is less than 2% [3]. Glomus tumors can occur in other areas, including the gastrointestinal tract, female reproductive tract, cardiovascular, and respiratory systems [4, 5, 6]. Glomus tumor of the respiratory tract is most common in the trachea and bronchial airway, and malignant glomus tumor originating in the lung is rare, with an estimated incidence of < 0.2% among primary lung neoplasms [7, 8]. We report a case of primary malignant pulmonary glomus tumor confirmed by pathological biopsy and immunohistochemistry. We review the relevant literature and highlight their imaging features.

## 2 Case Report

**FIGURE 1–3:**
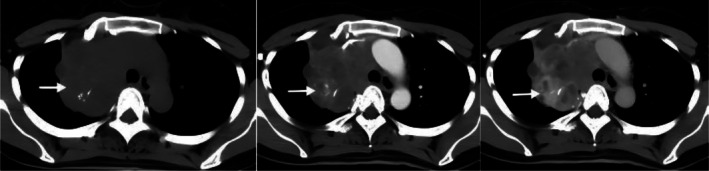
Initial chest CT. (1) Axial noncontrast image shows a large mass in the right upper lobe with speckled calcifications (→). (2) Arterial phase shows heterogeneous enhancement (→). (3) Venous phase shows progressive enhancement (→).

**FIGURE 4–6:**
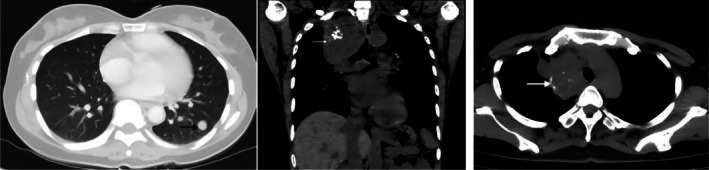
(4) CT image shows a metastatic nodule in the left lower lobe (→). (5) Follow‐up CT after anlotinib therapy shows reduction in the right upper lobe mass (→) and metastatic nodules (→).

The patient was a 41‐year‐old female. Cough, sputum, and chest pain for 2 years, accompanied by paroxysmal needlelike chest pain, accompanied by shortness of breath after activity. There has been no improvement in local treatment of pneumonia, repeated attacks. Six months ago, the above symptoms worsened, cough and sputum increased before 3 days, coughing a small amount of bright red blood sputum. No significant family or personal history of malignancy was reported. No significant family or personal history of malignancy was reported. Chest CT showed: Soft tissue density mass shadow was seen in the upper lobe of the right lung, with a maximum cross section of about 8.8 × 6.8 cm, multiple speckled calcification foci were seen inside, the boundary with the right mediastinum was unclear, the trachea was slightly compressed, the bronchus in the upper lobe of the right lung was narrowed and blocked, the enhanced scan was uneven and enhanced, the superior vena cava was compressed (Figures 1–3), low‐density shadow was seen in the upper lobe of the right lung, multiple nodules were seen in both lungs, and the larger one was located in the lower lobe of the left lung (Figure 4). The diameter is about 1.3 cm. Enlarged lymph nodes were seen in the mediastinum and right hilum, and the right upper pleura was thickened.

**FIGURE 7:**
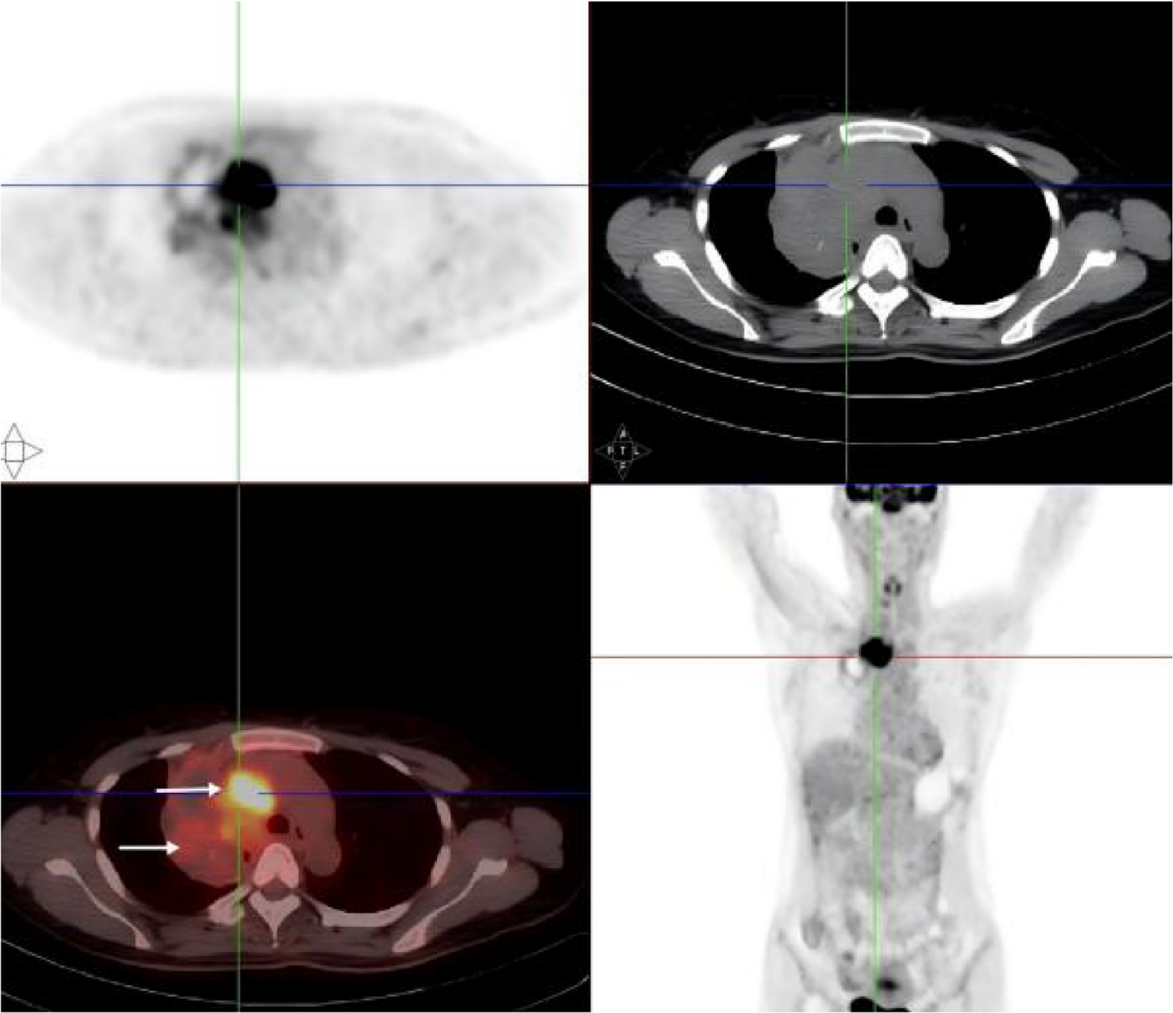
PET‐CT shows a mediastinal mass in the right superior lobe of the lung (→), with enlarged lymph nodes in the mediastinum (→) and right hilum of the lung, and increased FDG metabolism in the lesion.

^18^F‐FDG PET‐CT: A mass was found beside the mediastinum of the superior lobe of the right lung. The density of 8.4 × 6.5 cm is not uniform; there are multiple low‐density shadows and scattered calcification foci in it, and the boundary with the mediastinum is not clear. In clear, multiple high metabolic concentration foci of FDG could be seen in the lesion; the larger one was located near the aortic arch, and the range was about 4.1 × 2.5 cm. SUV_max_ is about 11.2 (Figure 7). Multiple nodules could be seen in both lungs; the larger one was located in the lower lobe of the left lung with a size of about 1.5 × 1.1 cm. FDG metabolism was increased in the nodules of the lower lobe of the left lung with a SUV_max_ value of 6.5. There were enlarged lymph nodes in the mediastinum and right lung hilum, increased FDG metabolism, and SUV_max_ was 4.3. The left adrenal gland is visible with a diameter of approx. No increase in FDG metabolism was observed in 0.5‐cm nodules.

**FIGURE 8:**
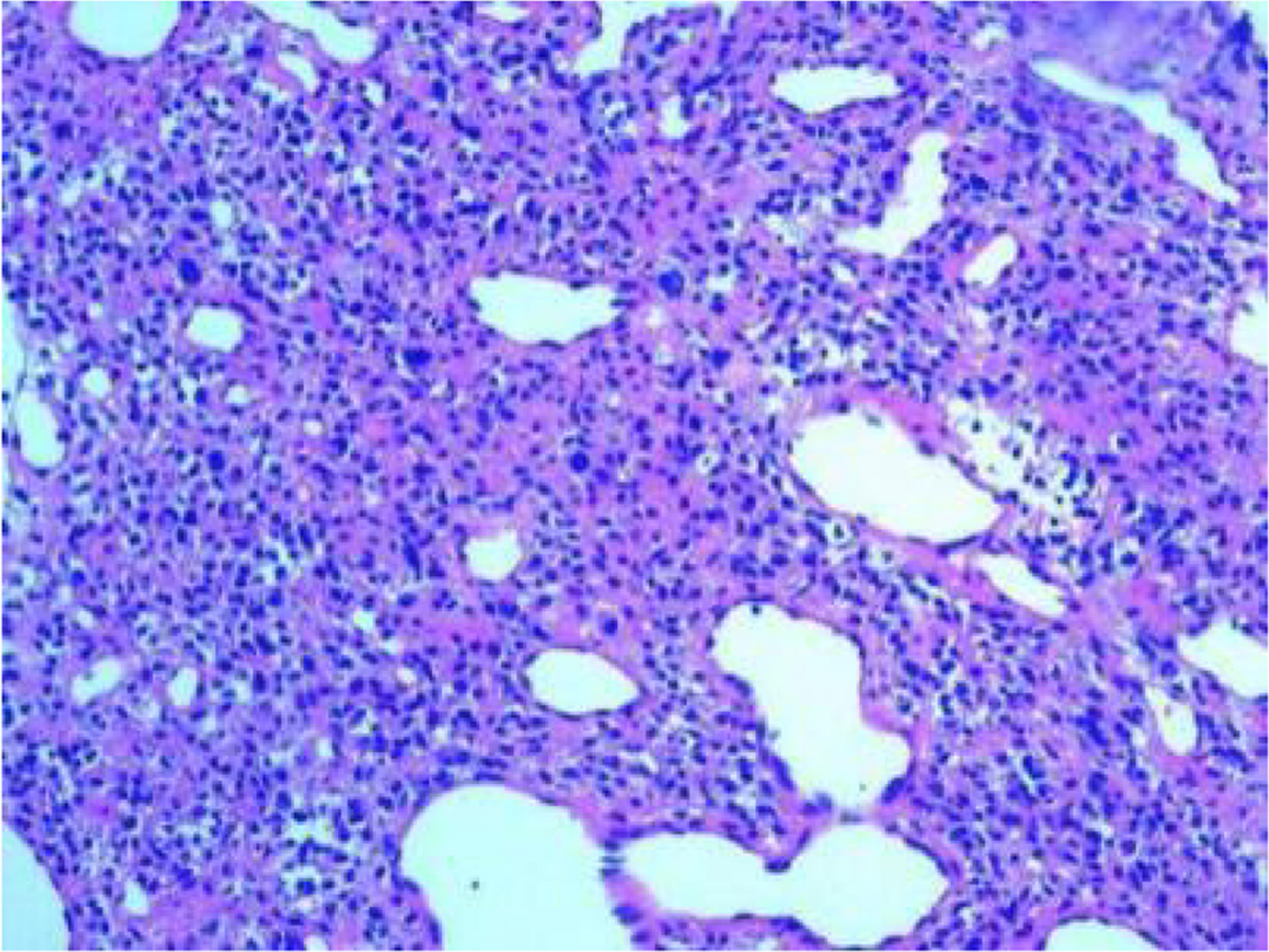
The tumor were polymorphic with obvious nuclear atypia and pathological nuclear division. H&E stain, original magnification x200.

**FIGURE 9:**
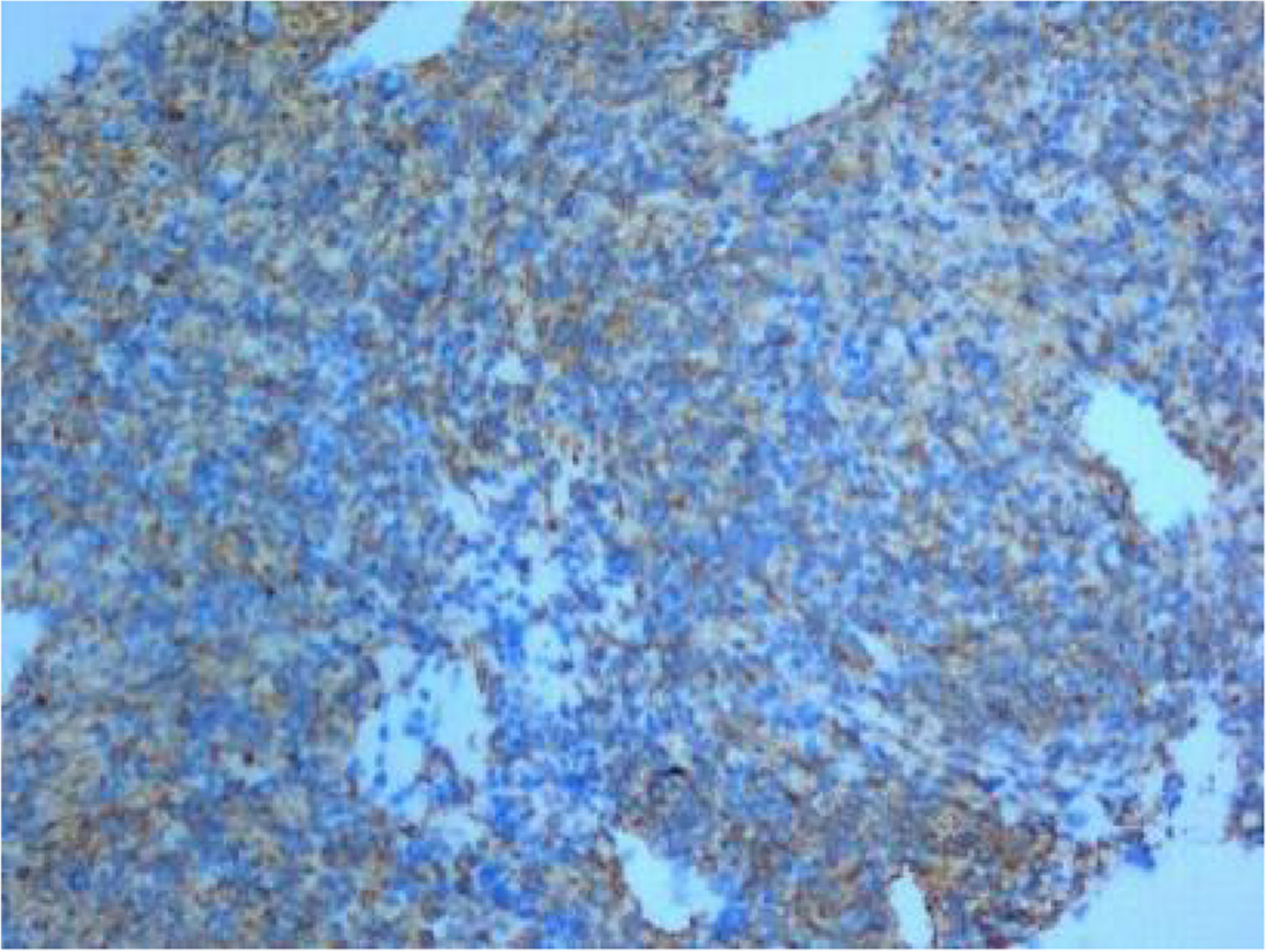
The tumor cells were positive for *α*‐SMA. EnVision method, original magnification x200.

Biopsy of the right lung showed that the tumor was rich in thin‐walled blood vessels, with irregular lumen, visible branches and swelling. Tumor cells grow diffusely around blood vessels. Tumor cell boundary is clear, round or polygonal, partial cell fusiform, abundant cytoplasm, acidophilic granulosis or light staining; nuclear atypia is more obvious, some have nucleoli, seen in the nucleus. Inclusion body, nuclear division (4/50HPF) (Figures 8 and 9), and see pathological nuclear division (figure). Immunohistochemical staining: swelling. Tumor cells SAM, caldesmon and vimentin (diffuse +), Syn (partial +), CD34 (blood tube +), Ki‐67 (20%), EMA, CK, CgA, S‐100, Desmin, LCA, MelanA, HMB45 both (−). Pathological diagnosis: malignant glomus tumor of the right lung.

Due to metastasis of both lungs and mediastinal lymph nodes, the patient was reluctant to undergo surgery and received chemotherapy. Follow up 6 months later. On chest CT, the mass and intrapulmonary metastases in the upper lobe of the right lung were larger than before. Amlotinib hydrochloride was then self‐administered. After targeted therapy, chest CT at 17, 28, and 36 months follow‐up showed that the right lung mass and intrapulmonary metastasis were smaller than before (Figures 4–6). There were no new metastases.

## 3 Discussion

Glomus tumor originates from glomus cells, which are a kind of mutated smooth muscle cells located at arteriovenous anastomosis [9] and are distributed under the nail bed of the finger (toe) at the end of limbs. Therefore, glomus tumor is most likely to occur in the palm, wrist, forearm, and foot and is mostly benign and can also be found in the nasal cavity, trachea, lung, thyroid gland, esophagus, stomach, colon, rectum, and other parts [1, 3, 4, 5, 6, 7, 8]. Due to the lack of angiospheroids, glomus tumors originating in the lungs are very rare. Up to now, more than 40 cases of pulmonary glomus tumors have been reported in Chinese and foreign literature [10, 11, 12]. The age of onset ranges from 9 to 83 years, mostly in middle and old age, and the proportion of men and women is similar [11, 12]. There are no clinical characteristics; The common symptoms are cough, sputum, hemoptysis, chest pain, dyspnea, and so on.

According to the 2020 WHO Classification of Soft Tissue Tumours [13], glomus tumors are classified into benign glomus tumors, glomus tumors with uncertain malignant potential, malignant glomus tumors, and glomus tumors. The diagnostic criteria for malignant glomus tumors are as follows: (1) significant nuclear atypia and any level of nuclear division; and (2) atypical nuclear division. Those who do not meet the diagnostic criteria for malignant lesions and have at least one nuclear atypia should be diagnosed as hemangioma with uncertain malignant potential. Immunohistochemical markers showed that the tumor cells of hemangioma diffuse expression of SMA, muscle‐specific actin, vimentin, caldesmon, calponin, and type IV collagen and do not express S‐100 and cytokeratin [14]. Slightly different from soft tissue glomus tumors, malignant tumors of lung and trachea are relatively high. It has been reported in the literature [11, 12] that the majority of primary glomus tumors of lung and trachea are benign, accounting for about 74%, followed by malignant glomus tumors (about 21%) and glomus tumors with uncertain malignant potential (about 5%). The reason may be related to the deep location of lung or trachea tumors; if the patient has no symptoms or regular physical examination, the tumor is not easy to be detected early.

CT findings of pulmonary hemangioma showed nodules or masses, occasionally coin‐like nodules, ranging in size from 1.0 to 9.7 cm, with uneven density and no calcification or cavity. Enhanced CT showed uneven peripheral enhancement, and the central enhancement was not obvious. Enhanced CT reflected the distribution of peripheral blood vessels of the tumor [10, 11, 12, 15, 16, 17, 18, 19, 20, 21]. Cunningham et al. [10] believe that benign and malignant pulmonary glomus tumors have no specificity in imaging, and the sizes of benign and malignant tumors overlap considerably. The tumors with the largest diameter of 9.7 cm are pathologically benign [11, 12]. MRI findings of glomus tumor are rarely reported, showing equal signal on T_1_WI and high signal on T_2_WI, and high signal in the central area on both T_1_ and T_2_ images. Enhanced MRI showed significant peripheral enhancement in the early stage, gradually extending to the central part of the tumor, and there was no enhancement in the center of the tumor [19]. It has been reported in the literature that FDG PET in pulmonary glomus tumors presents as round or irregular soft tissue masses with low to moderate intensity FDG accumulation [10, 22], with SUVmax ranging from 4.5 to 5.2, and malignant glomus tumors may also present with low to moderate uptake.

The imaging characteristics of pulmonary malignant glomus tumor are lacking. The differential diagnosis includes carcinoid and hemangiopericytoma/solitary fibroma, smooth muscle tumor (especially epithelioid leiomyoma), primitive neuroectodermal tumor (PNET), paraganglioma, and metastatic tumor [23]. Histological and immunohistochemical staining can be used to distinguish these tumors effectively.

Surgical treatment is the first choice for pulmonary hemangioma. Benign hemangioma has a good prognosis. No postoperative recurrence and malignant transformation have been reported. The prognosis of malignant pulmonary vascular balls is still unclear, and distant metastasis is the main cause of poor prognosis and death. Metastasis usually occurs 3 to 4 years after surgery, with a metastasis rate of 31.2% to 38.0% [24]. DeCocker et al. indicated that sublobectomy by wedge, anatomical segmentectomy, or sleeve resection is the preferred treatment for malignant pulmonary glomus tumor [7, 25, 26], which can be combined with mediastinal lymph node dissection and postoperative chemotherapy. The role of chemotherapy and targeted therapy is not well‐established. In this case, anlotinib showed clinical benefit, though its mechanism in glomus tumors remains unclear.

## 4 Conclusion

Primary malignant pulmonary glomus tumor is a rare malignancy with nonspecific clinical and imaging features. Diagnosis relies on histopathology and immunohistochemistry. Surgical resection is curative for localized disease, while the role of systemic therapy requires further study. Long‐term prognosis remains uncertain, particularly in metastatic cases.

## Author Contributions

**Shishi Luo:** writing – original draft. Xiaoyan Lei: writing – original draft. **Tianxu Fu:** methodology, supervision, writing – review and editing. **Fujin Liu:** methodology, supervision, writing – review and editing. **Zhenping Wang:** funding acquisition, writing – review and editing. Shishi Luo and Xiaoyan Lei have contributed equally to this work.

## Funding

This study was supported by the Joint Program on Health Science & Technology Innovation of Hainan Province (WSJK2025MS198, SQ2023WSJK0224) and project supported by the Hainan Province Clinical Medical Center of China (LY21H080005).

## Ethics Statement

The studies involving humans were approved by the Medical Research Ethics Committee of Hainan General Hospital. The studies were conducted in accordance with the local legislation and institutional requirements. The participants provided their written informed consent to participate in this study. Written informed consent was obtained from the individual(s) for the publication of any potentially identifiable images or data included in this article. Written informed consent was obtained from the participant/patient(s) for the publication of this case report.

## Conflicts of Interest

The authors declare no conflicts of interest.

## Data Availability

The data that support the findings of this study are available on request from the corresponding author. The data are not publicly available due to privacy or ethical restrictions.
